# The mechanism of assortative mating for educational attainment: a study of Finnish and Dutch twins and their spouses

**DOI:** 10.3389/fgene.2023.1150697

**Published:** 2023-06-14

**Authors:** Bodine M. A. Gonggrijp, Karri Silventoinen, Conor V. Dolan, Dorret I. Boomsma, Jaakko Kaprio, Gonneke Willemsen

**Affiliations:** ^1^ Netherlands Institute for the Study of Crime and Law Enforcement (NSCR), Amsterdam, Netherlands; ^2^ Department of Biological Psychology, Faculty of Behavioural and Movement Sciences, VU Amsterdam, Amsterdam, Netherlands, Netherlands; ^3^ Faculty of Social Sciences, University of Helsinki, Helsinki, Uusimaa, Finland; ^4^ Department of Public Health, Faculty of Medicine, University of Helsinki, Helsinki, Uusimaa, Finland; ^5^ Amsterdam Public Health Research Institute, VU Medical Center, Amsterdam, Netherlands; ^6^ Institute for Molecular Medicine Finland, Helsinki Institute of Life Science, University of Helsinki, Helsinki, Uusimaa, Finland

**Keywords:** assortative mating, education, twins, social homogamy, phenotypic assortment

## Abstract

**Introduction:** Assortative mating refers describes a phenomenon in which individuals with similar phenotypic traits are more likely to mate and reproduce with each other; i.e. assortative mating occurs when individuals choose partners based on their similarity or dissimilarity in certain traits.to patterns of non-random mating of spouses leading to phenotypic resemblance. There are various theories about the its underlying mechanisms, which have different genetic consequences.

**Methods:** We analyzed examined two possible mechanisms underlying assortative mating – phenotypic assortment and social homogamy – for educational attainment in two countries utilizing data of mono- and dizygotic twins and their spouses (1,451 Finnish and 1,616 Dutch twin-spouse pairs).

**Results:** The spousal correlations were 0.51 in Finland and 0.45 in the Netherlands, to which phenotypic assortment contributed 0.35 and 0.30, and social homogamy 0.16 and 0.15, respectively.

**Conclusion:** Both social homogamy and phenotypic assortment are important processes in spouse selection in Finland and the Netherlands. In both countries, phenotypic assortment contributes to a greater degree to the similarity of spouses than social homogamy.

## Introduction

Assortative mating describes the phenomenon that partners tend to resemble, for a particular trait, or sets of traits, each other more than when mating would occur completely at random. The degree to which partners are alike has been studied extensively during the past decades showing that assortative mating is important in physical characteristics, lifestyle, intelligence, and educational attainment (Schwartz, 2013; Luo, 2017). Prior research has indicated that for educational assortment spousal correlations range from 0.37 to 0.66 (Abdellaoui et al., 2015; Hur, 2016; Robinson et al., 2017; Sherlock et al., 2017; Tambs, Sundet, and Berg, 1993; Torvik et al., 2021; Zietsch et al., 2012). Over the past 50 years, the level of educational assortment has steadily increased in the United States, Denmark, Norway, the United Kingdom, and Germany (Eika et al., 2019; Mare, 2016). This increase has also been seen in other countries worldwide (Permanyer et al., 2019).

Assortative mating for educational attainment can have significant consequences for society. When educational attainment is more similar in partners, there is an increase in variance in educational attainment in the population, until equilibrium is reached (Falconer and Mackay, 1996) and in offspring being more likely to have two parents with either low or high levels of education, rather than a mix of both, which can lead to greater income inequality and social stratification (Schwartz, 2013; Greenwood, et al., 2014; Eika et al., 2019; Ciscato and Weber, 2020). Depending on the mechanisms that underlie assortative mating for educational attainment, it may also have genetic consequences. Phenotypic assortment implies that the choice of a partner is based upon similarity in a partner’s observable or known characteristics. In so far as such characteristics are subject to genetic effects, this will lead to increased genetic similarity among parent–offspring, and sibling pairs, and an increase in the genetic variation until equilibrium is reached (Falconer, 1960; Heath and Eaves, 1985). Because educational attainment is a heritable trait (i.e., individual differences in educational attainment are associated with genetic individual differences; Silventoinen et al., 2020), phenotypic assortment for educational attainment implies indirect genetic assortment and a genetic correlation between spouses. It also increases the additive genetic correlation of DZ twins and full siblings above 0.50, i.e., the expected correlation under random mating. The incorrect assumption of random mating may thus bias the estimates of genetic and environmental variance components in the classical twin design (Eaves, 1979; Heath and Eaves. 1985). A recent study by Torvik et al. (2021) that looked at the genetic similarities between Norwegian spouses, siblings, and in-laws for educational attainment, depression, and height found evidence of genetic similarity between spouses for educational attainment (genetic correlation, rg = 0.37). In addition, the genetic correlation between full sibs based on common genetic variants related to educational attainment was 0.68, which suggests the presence of assortative mating. Their overall findings suggested that assortative mating has influenced the genetic structure of extended families for several generations. However, an earlier study in the United States found that the spousal genetic correlation remained stable over the last decades in spite of increased phenotypic correlation (Conley et al., 2016). This suggests that, while it likely plays a role, phenotypic assortment may not be the only process underlying assortative mating for educational attainment.

In addition to phenotypic assortment, at least two other processes can contribute to assortative mating: convergence and social homogamy. In the case of convergence, also referred to as marital interaction (van Grootheest et al., 2008), spouses become more similar to each other due to the environment they share. If convergence is present, the similarity of partners should increase the longer they live together. No evidence has been found for convergence with respect to educational attainment, as the degree of spousal similarity does not correlate with the length of the relationship (Mascie-Taylor and Vandenberg, 1988; Caspi and Herbener, 1993; Watson et al., 2004; Zietsch et al., 2012). This is understandable as most stable or long-term partner relationships tend to be established when formal education has been completed. In many developed countries (such as Finland and Netherlands), first marriage generally occurs after age 25, while education is often completed by that age. In the case of social homogamy, partners choose each other from amongst people living in the same social environments. As such, social homogamy may not have genetic consequences (Falconer, 1960; Eaves et al., 1989). To make a distinction between the different processes of assortative mating, we can apply genetically informative designs. Analyzing data on twins and their spouses allows for disentangling the processes of social homogamy and phenotypic assortment by utilizing the different genetic similarities of monozygotic (MZ) and dizygotic (DZ) twins (Eaves et al., 1978; Heath and Eaves, 1985). In this study, we apply this design to data of twins and their spouses in Finland and Netherlands. These countries have a similar schooling system, with education being universally available and (mostly) publicly funded, and the number of years needed to obtain an educational degree is similar (NUFFIC, 2020).

## Materials and methods

### Participants

#### Finnish twin cohort (FTC)

The Finnish sample was collected in the fifth wave of data collection of the FinnTwin16 study, which took place between 2010–2012 (Kaidesoja et al., 2019). The twins, born between 1975 and 1979, received an internet survey with questions about health, lifestyle, and personality. The twins were asked to invite their intimate partners to participate. Twins identified their partners themselves and provided information concerning the length and nature of the relationship.

Data were available from 6,115 participants. Due to the setup of the Finnish data collection, the vast majority (95%) of the participants were 30 years or older. Data from 308 spouse pairs under the age of 30 were excluded from the analyses. Twin pairs were removed because zygosity was missing or unknown. Twin zygosity was determined based on self-report of physical similarity and, if necessary, by parental report. This combined method has high validity in this cohort (Jelenkovic et al., 2011). This resulted in a total sample of 3,993 twins, and 1,607 spouses of which 1,452 were complete twin-spouse pair responses. The sample included 1,301 MZ, 1,284 same-sex dizygotic (SSDZ) and 1,408 opposite-sex dizygotic (OSDZ) twins. There were 794 families, with one twin-spouse response, and 329 families that included a twin-spouse response for both twins (Table 1). The mean age for the male twins was 34.1 (SD 1.14, range 31–38 years), and for the female twins 34.0 (SD 1.15, range 31–38 years). The mean age for the male spouses was 36.4 years (SD 4.2, range 30–61 years) and for the female spouses 33.7 years (SD 3.1, range 30–52 years).

**TABLE 1:** The family configuration of the data.

	Finnish			Dutch		
	No data from spouse	Data available of one twin-spouse pair	Data available of both twin-spouse pairs	No data from spouse	Data available of one twin-spouse pair	Data available of both twin-spouse pairs
MZ						
Families yielding a single twin	133	64	-	378	259	-
Families yielding a twin pair	268	153	128	263	214	156
SSDZ						
Families yielding a single twin	182	97	-	277	183	-
Families yielding a twin pair	228	163	109	105	119	86
OSDZ						
Families yielding a single twin	224	138	-	282	195	
Families yielding a twin pair	245	179	92	66	76	43
Total	1,280	794	329	1,371	1,046	285

Note: MZ, monozygotic twins; SSDZ , same-sex dizygotic twins; OSDZ, opposite-sex dizygotic twins.

### Netherlands twin register (NTR)

The NTR includes information about the health, lifestyle, and personality in adult twins and their family members (Ligthart et al., 2019). The registered families receive surveys on health and lifestyle every two or 3 years. Nearly all surveys included comparable questions on completed and current education. For both spouses and twins, data from all surveys were compared and checked for discrepancies, and a single measure for educational attainment was obtained. Twins identified their partner themselves as “the partner with whom you share a lasting and stable relationship (like a marriage).” Spousal data concerning educational level were based on self-report and/or the report of the twin (i.e., the twin reported the educational level of the spouse). If spousal self-report was absent, data reported by the twins in the most recent survey was used (54% of the spousal data). Spousal self-report was compared to the educational level reported by the twin, which was concordant for 79% (N = 617). Discrepancies were due to a higher spousal self-report when comparing to twin report in 14% of the cases while in 7% of the cases this was due to lower education according to self-report.

Data were available for 11,077 twins born between 1909 and 1992. All participants younger than 30 years old were excluded from the analyses (N = 3,697) to ensure comparability with the Finnish sample. 101 twin pairs with missing or unknown zygosity were removed. Twin zygosity was determined by genotyping or by self and parental report concerning the physical resemblance of the twins or confusion by other family members and peers (Ligthart et al., 2019). Lastly, all twins born before 1965 and after 1989 were excluded from the analyses to make the data more comparable with the Finnish data (N = 1,958). This resulted in a total sample of 5,839 twins, 1,241 spouses and 1,616 twin-spouse pairs. The sample included 1,936 MZ, 1,091 SSDZ, and 854 OSDZ twins. There were 1,046 families in which data were available for one twin-spouse pair, and 285 families in which data was available for both twin-spouse pairs (Table 1). The mean age of the twins was 37.6 years (SD 4.95, range 30–61 years), the mean age of the male spouses was 38.2 years (SD 5.90, range 30–75 years) and of the female spouses 36.6 years (SD 4.93, range 30–53 years).

### Educational attainment

In Finland and Netherlands, education is compulsory until age 16. After 16, education continues either on an academic tract to a high-school diploma and possible university studies, or on a vocational school tract, and later at universities of applied sciences. During their education, individuals may change track. For both countries, educational attainment was based on the highest educational level obtained. In Finland, the following levels were distinguished: 1) Primary education or junior high school; 2) Vocational school or comparable; 3) College level or comparable; 4) University of applied sciences or higher vocational schooling; and 5) College or university. Educational attainment was transformed to the number of years of education by using information about the schools the participants had attended, and the degrees they had obtained or were obtained at the time they completed the survey, as described in Table 2. If participants reported being engaged in education (at the time of the survey), they received the number of years indicated in Table 2 minus 1 year.

**TABLE 2:** Number of years attained per completed educational level.

Finland		Netherlands	
Educational level	Total number of years	Educational level	Total number of years
Junior high school	9	Primary education	6
Vocational School	10	Lower vocational schooling/Lower secondary schooling (general)	10
College level/Senior High School	12	Intermediate vocational schooling/higher secondary schooling	13
University of Applied Sciences	15	Higher vocational schooling	15
College or University	16	University	16

In Netherlands, educational attainment was available as a composite measure based on all survey data available. The answer categories varied per survey and were recoded into seven categories: 1 = primary school only; 2 = lower vocational schooling; 3 = lower secondary schooling (general); 4 = intermediate vocational schooling; 5 = intermediate/higher secondary schooling (general); 6 = higher vocational schooling; and 7 = university. If a participant indicated not to have completed the highest schooling, then the next lower educational attainment level was used. The data were harmonized to same categories as in Finland by combining lower vocational schooling with lower secondary schooling (general) to category two; vocational school or comparable and combining intermediate vocational schooling with intermediate/higher secondary schooling (general) to category 3; college level or comparable. The number of years of the five categories was coded as described in Table 2.

### Statistical modeling

**FIGURE 1:**
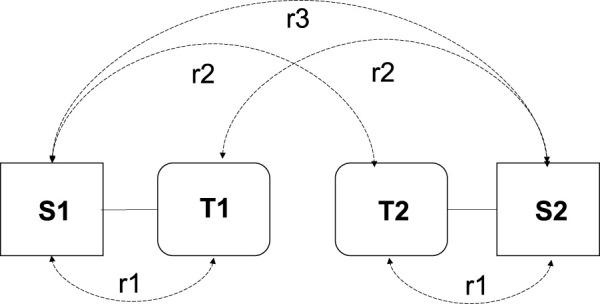
Schematic representation of twins and their spouses including the correlations. T, twin; S, spouse; r1, twin-spouse correlation; r2, cotwin-spouse correlation and r3, spousal correlation. This figure is derived from van Grootheest et al., 2008.

The correlations between twins and their spouses (ɾ1), twins and the spouse of their co-twin (ɾ2), and spouses of both twins (ɾ3) provide an initial indication of the presence of phenotypic assortment or social homogamy, as illustrated in Figure 1. If phenotypic assortment is the sole assortment process, we expect the following rank order of the correlations: ɾ1 > ɾ2 > ɾ3. In the case of purely social homogamy, we expect equal correlations, i.e., ɾ1 = ɾ2 = ɾ3 (Reynolds et al., 2006). If the phenotype is subject to genetic effects, the correlations of twins and their co-twins spouses and the spousal correlations will be greater for the MZ twins than for the DZ twins, i.e., ɾ1MZ > ɾ1DZ and ɾ2MZ> ɾ2DZ (Reynolds et al., 2006).

**FIGURE 2:**
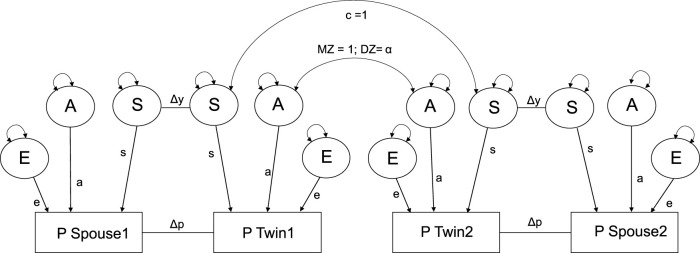
Spouse selection on phenotype and social background. a, genetic path regression; s, social background path regression, which includes shared environmental influences (c); e, environmental path regression; ΔY, selection based on social background environment; Δp, selection based on phenotype. The A factors are constrained to 1.0 for MZ pairs and *α*, DZ genetic similarity *α*, 0.5 (1 + Δp) for DZ pairs and the C factors are constrained to one for both MZ and DZ pairs. This model is derived from Reynolds et al., 2006.

Figure 2 represents an adjusted version of the twin-spouse model of Reynolds et al. (2006). Details on the implementation of different twin-spouse models with power calculations can be found in the Supplementary Material. We found that including both shared environmental (C) and social background environment (S) leads to empirical under-identification. Therefore, the shared environment (C) was dropped from the model and will be fully absorbed by the social background environmental parameter (S). The proposed model in Figure 2 thus allows for the decomposition of additive genetic (A), social environmental (S), and non-shared environmental (E) variance, while simultaneously parameterizing both phenotypic assortment (Δp) and social homogamy (Δy). Phenotypic assortment is modeled by the delta path Δp, i.e., by the direct associations between the phenotype of twins and their spouses. Social homogamy is modeled by the delta path Δy, i.e., by direct associations between the social background factors (s). In both MZ and DZ twins raised together, the correlation of social background factors is 1 as it is part of their common environment. The effect of sex was accounted for by including the main effect of sex on the phenotype.

We note that persistent phenotypic assortment results in an increase in genotype variance to an equilibrium value (Falconer, 1960). A second consequence is that it induces genotype-environment correlation between spouses. In addition, cultural transmission (i.e., the EA of the parents impinges on the environment of the offspring—a plausible hypothesis), results in within person genotype environment correlation (Fulker, 1988). In our models, the genotype-environment correlation follows from the delta path (Δp). In contrast to phenotypic assortment, which has these indirect effects on the distribution of the genotypic and environmental factors, social homogamy operates at the (latent) environmental level, and so does not have these effects (Heath and Eaves, 1985; Eaves et al., 1989). In the current research we assume that phenotypic assortment process is in equilibrium.

The full twin-spouse model, which included the A, S, E, Δp, and Δy parameters, was fitted to the data by maximum likelihood estimation using the R library OpenMx version 3.6.1 (Neale et al., 2016). In addition, several more parsimonious sub-models (nested under the full model) were fitted. Statistical testing was based on likelihood ratio tests. Minus twice the difference in the log-likelihood function values of the competing models follow a χ2-distribution if the constraints associated with the more parsimonious model are correct. The degrees of freedom of the χ2-distribution equals the difference in the number of estimated parameters of the competing models. We adopted an alpha of 0.05 in conducting the tests. In the sub-models, the paths Δp and Δy were constrained to equal zero simultaneously and separately to determine if phenotypic assortment, social homogamy, or both accounted for the assortative mating for educational attainment.

## Results

### Phenotypic analyses

Descriptive statistics of educational years in the Finnish and Dutch cohort are given in Table 3 (the descriptive statistics of educational level can be found in Supplementary Table S1). In both cohorts, more female than male twins participated, and more spouses of female twins than spouses of male twins participated. Looking at the educational level in Finland, women had a higher average education compared to men in twins [t(3573.38) = −7.87, *p* < 0.001] and spouses [t(1588.66) = −5.23, *p* < 0.001]. In Netherlands, educational level was significantly higher for the female spouses [t(930.66) = −5.44, *p* < 0.001], but no significant differences were found between male and female twins.

**TABLE 3:** Number of educational years, mean and SD for the Finnish and Dutch cohort divided by men and women.

		Educational years Finland			Educational years Netherlands		
		N	Mean	SD	N	Mean	SD
Men							
	Twin	1741	13.09^a^	2.52	1148	14.09	1.92
	Spouse	895	13.27^b^	2.52	1189	13.56^c^	2.24
Women							
	Twin	2252	13.70^a^	2.33	2496	13.36	2.31
	Spouse	712	13.89^b^	2.22	432	14.14^c^	1.82

a, b, c statistical significant difference between groups *p* < 0.001.

Table 4 presents educational attainment correlations for twins, twin and own spouse, cotwin and spouse and spouse with spouse. The twin correlations indicate heritability, as MZ correlations were higher in MZ twins. DZ correlations were greater than half of the MZ correlations, which suggests the presence of shared environmental influences or assortative mating in the parents. Assortative mating for educational attainment was evident in the twin-spouse correlations in both cohorts, as the correlations between twins and their own spouses were 0.48 and 0.45 for EA. In both the Finnish and Dutch cohorts, the rank order of the average correlations between twins and their spouses was ɾ1> ɾ2 > ɾ3, thus providing evidence for phenotypic assortment. However, this pattern was found in most, but not all zygosity groups (see Supplementary Table S2). For the Finnish DZm twins and for the Dutch MZm, DZm, and DZf twins the pattern of correlations was ɾ1 > ɾ2 < ɾ3, which suggests both phenotypic assortment and social homogamy.

**TABLE 4:** Correlations between twins, twin and own spouse, co twin and spouse and spouse with spouse with 95% confidence intervals.

	Finland				Netherlands			
	All twins	MZ	DZ	DOS	All twins	MZ	DZ	DOS
	(95% CI)	(95% CI)	(95% CI)	(95% CI)	(95% CI)	(95% CI)	(95% CI)	(95% CI)
ɾtw1– ɾtw2	0.53**	0.70**	0.54**	0.36**	0.60**	0.74**	0.43**	0.41**
	(0.50– 0.57)	(0.66– 0.74)	(0.47– 0.60)	(0.38– 0.43)	(0.57– 0.64)	(0.70– 0.77)	(0.34– 0.52)	(0.24– 0.56)
ɾtw– ɾsp	0.48**	0.44**	0.49**	0.50**	0.45**	0.45**	0.43**	0.47**
	(0.43– 0.51)	(0.37– 0.51)	(0.41– 0.55)	(0.43– 0.56)	(0.41– 0.49)	(0.39– 0.50)	(0.36– 0.50)	(0.35– 0.58)
ɾcotw– ɾsp	0.34**	0.39**	0.40**	0.25**	0.37**	0.38**	0.35**	0.38**
	(0.29– 0.39)	(0.30– 0.46)	(0.31– 0.48)	(0.16– 0.34)	(0.32– 0.42)	(0.31– 0.45)	(0.25– 0.44)	(0.26– 0.50)
ɾsp1–ɾsp2	0.30**	0.32**	0.45**	0.13**	0.33**	0.34**	0.43**	−0.003
	(0.20– 0.39)	(0.15– 0.46)	(0.29– 0.58)	(−0.06– 0.31)	(0.24– 0.42)	(0.21– 0.45)	(0.26– 0.58)	(−0.38– 0.37)

**Correlations are significant at the 0.01 level (2-tailed). MZ, monozygotic twin pairs; DZ, dizygotic twin pairs; DOS, dizygotic opposite-sex twin pairs.

### Model-fitting

#### Finland

The model fit statistics for the full and nested models are available in Supplementary Table S3. First, a main effect of sex was found, i.e., b = 0.64 (*p* < 0.01), with women, on average, tend to have 0.64 more years of education compared to men. Simultaneously and separately setting the parameters for phenotypic assortment (Δp) and social homogamy (Δy) to zero consistently resulted in significant likelihood ratio tests and hence worse fit of the model to the data (Supplementary Table S3). This means that both phenotypic assortment and social homogamy contributed to the spousal correlation for educational attainment. Table 5 presents the decomposition of the proportions of the variance in educational attainment explained by genetic, shared environmental, and unique environmental factors, and the proportions of the spousal correlation explained by social homogamy and phenotypic assortment. The raw parameter estimates with 95% confidence intervals for the model including both estimated Δp, and Δy are given in Supplementary Table S4. Genetic, shared environmental and non-shared environmental influences accounted for 55%, 16%, and 29%, respectively, of the variance in educational attainment.

**TABLE 5:** The proportions of the variation in educational attainment explained by genetic, shared environmental and unique environmental factors and the proportions of the spousal correlation explained by social homogamy and phenotypic assortment.

	Finland			Netherlands		
		95% CI			95% CI	
	Proportion	LB	UB	Proportion	LB	UB
Variation educational attainment						
h2	0.55	0.44	0.66	0.66	0.52	0.77
e2	0.29	0.26	0.33	0.21	0.19	0.24
s2	0.16	0.04	0.24	0.13	0.02	0.26
Spousal correlation						
Δy	0.16	0.03	0.17	0.15	0.02	-
Δp	0.35	0.27	0.43	0.30	0.20	0.40

h2, additive genetic factors; e2, unique environmental factors; s2, social environmental factors; Δy, social homogamy; Δp, phenotypic assortment. LB, lower bound; UB, upper bound.

The spousal correlation was estimated at 0.51, and is a function of both phenotypic assortment (Δp = 0.35) and social homogamy. The contribution of social homogamy to the estimated spousal correlation is a function of the shared environmental effects (with variance denoted c2) and the direct effect of social background factors (Δy = 1.03). Specifically, the contribution of social homogamy equals c2*Δy = 0.3922*1.03 = 0.16. Thus, phenotypic assortment (0.35) contributes more to the similarity of spouses than social homogamy (0.16).

#### Netherlands

Comparable results were found in the Dutch cohort. Again, a sex effect was found (b = 0.20; *p* < 0.01), favoring the females. Dropping phenotypic assortment and social homogamy separately and simultaneously consistently resulted in significant likelihood ratio tests (Supplementary Table S3). Genetic effects accounted for 66% of the variance in educational attainment, shared environmental for 13% and non-shared environmental influences for 21%. The spousal correlation was estimated at 0.45, and is a function of the estimated phenotypic assortment (Δp = 0.30) and the contribution of social homogamy (c2*Δy = 0.3622*1.18 = 0.15). So, similarly to what was found in the Finnish cohort, phenotypic assortment contributed a greater degree to the similarity of spouses than social homogamy.

## Discussion

The aim of this study was to examine the mechanisms underlying spousal associations of educational attainment in Finland and Netherlands, two countries with comparable schooling systems and with a similarly high level of economic development. The results showed that the high degree of assortment for educational attainment was attributable to both phenotypic assortment and social homogamy, a finding that is consistent with previous studies. Reynolds et al. (2000) looked into the underlying mechanisms of assortative mating in educational attainment and fluid intelligence in Swedish twins and their spouses. They concluded that both social homogamy and phenotypic assortment processes contributed to assortment with respect to both phenotypes. Nagoshi et al. (1987) found significant social homogamy and phenotypic assortment for educational attainment in the Hawaii Family Study of Cognition among the Japanese and Chinese ancestries in the parent generation. With respect to effect sizes, our results suggest that phenotypic assortment contributes more than social homogamy to assortment. This is in line with findings reported by Nagoshi et al. (1987) and Vinkhuyzen et al. (2012), but not with those by Reynolds et al. (2000), who found that social homogamy and phenotypic assortment contributed equally to the spousal correlations of education attainment.

Our results revealed high twin spousal correlations in both Finland and Netherlands, which may be attributed to the increase in accessibility to higher education for the general population, particularly women. The majority of our participants received higher education (i.e., 54% Finland, 52% Netherlands), which may have driven the spousal correlation upwards, as seen for educational assortment worldwide (Eika et al., 2019; Mare, 2016; Permanyer et al., 2019).

In the classical twin design, estimates of genetic and environmental variances can be affected by the mechanisms of assortative mating. Failure to account for phenotypic assortment can result in an underestimation of shared environmental effects, while failure to account for social homogamy can lead to an overestimation of shared environmental effects (Falconer, 1960; Reynolds et al., 2000). In the current study, we observed a heritability of 66% in the Finnish cohort and of 55% in the Dutch cohort for educational attainment. Shared environmental influences accounted for 29% (Finland) and 21% (Netherlands) of the variance in educational attainment. When both phenotypic assortment and social homogamy were excluded from the model, genetic factors explained 53% of the variance in educational attainment and shared environmental 18% in Finland, *versus* 60% and 19% in Netherlands. We note that these variance estimates are all still well within the confidence intervals of the full model where assortative mating and social homogamy are accounted for. Omitting phenotypic assortment from the classical twin design leads to inflated estimates of shared environmental effects, while not modeling social homogamy leads to an underestimation of shared environmental effects (Falconer, 1960; Reynolds et al., 2000) and it is possible that their effects, at least partially, cancel each other out.

By analyzing data on the educational level of both twins and their spouses, this study was able to disentangle the two processes of assortative mating in two large samples. The year of birth of the twins did not differ more than 5 years in the Finnish twins and spanned 20 years in the Dutch twins. Educational attainment showed wide variation in the two samples meaning that all levels of educational attainment were well represented.

This study has also certain limitations. First, we focused on a single trait of educational attainment. We acknowledge that EA can be embedded in a network of correlated phenotypes, which may directly or indirectly contribute to the assortment for EA. Our conclusions should be interpreted within this context and future research may benefit from incorporating more phenotypes to assess the role of cross-trait assortative mating.

Secondly, we assumed an equilibrium model, where the process of spousal assortment has remained constant across generations. We acknowledge that this is likely violated to some degree, as educational systems have changed, especially the accessibility for women. In both Netherlands and Finland, the proportion of the population with tertiary education has been steadily increasing over the years, with around 50% of the Dutch and 45% of the Finnish population aged 25–64 having completed tertiary education in 2020 (Eurostat, 2022). Hence, the role of assortment processes is likely to differ across birth cohorts.

Lastly, while looking at the underlying mechanisms of assortative mating, the length of the relationship between the twins and spouses was not considered. However, the literature contains little evidence for convergence (Mascie-Taylor and Vandenberg, 1988; Caspi and Herbener, 1993; Watson et al., 2004; Zietsch et al., 2012). When looking at the correlation between the Dutch twins and their spouses as a function of their birth year, where it is likely that the older the participants are, the longer they are together, we saw no differences in spousal correlations, indicating that convergence does not contribute to assortment for educational attainment. Moreover, marriage on average occurs at an age after most, if not all, of formal education has been completed. Convergence may still occur if the pairs with the greatest differences in education separate more often than the pairs with similar education.

In conclusion, both social homogamy and phenotypic assortment are important processes in spouse selection in Finland and Netherlands with comparable educational systems, indicating that the underlying processes seem to be similar. Our results suggest that phenotypic assortment contributes to a greater degree to the similarity of spouses than social homogamy.

## Data Availability

The data analyzed in this study is subject to the following licenses/restrictions: datset can only be obtained by applying for the dataset at Netherlands Twin Register (NTR) and the Finnish Twin Register. Requests to access these datasets should be directed to https://ntr-data-request.psy.vu.nl/ and https://thl.fi/en/web/thl-biobank/for-researchers/application-process.
