# Supplementary material for: The mechanism of assortative mating for educational attainment: a study of Finnish and Dutch twins and their spouses
Source: Front Genet. 2023 Jun 14;14:1150697. doi: 10.3389/fgene.2023.1150697 (PMC10311485; doi:10.3389/fgene.2023.1150697)
Supplement: Supplementary file 1 [file DataSheet4.docx]

Supplementary Material

The mechanism of assortative mating for educational attainment: a study of Finnish and Dutch twins and their spouses

Bodine Gonggrijp*, K. Silventoinen, C.V. Dolan, D. Boomsmaa, J. Kaprio & G. Willemsen.

*** Correspondence:** Corresponding Author: b.m.a.gonggrijp@vu.nl

# Supplementary Figures and Tables 3

| Supplementary Table 3. *Model fit results for all the saturated models, divided per country. The best fitted model is printed in bold.* | | | | | | | | | | | | | | |
| --- | --- | --- | --- | --- | --- | --- | --- | --- | --- | --- | --- | --- | --- | --- |
| Model | Test | Versus | LL2 | *df* | AIC | Δ*df* | *p* | h | c | e | Δy | Δp | bsex |  |
| Finland | | | | | | | | | | | | | | |
| 0 | **Full model: A; C; E; dy, dp** | **-** | **24204.67** | **5490** | **13224.67** |  |  | **.74** | **.39** | **.54** | **1.03** | **.35** | **.64** |  |
| 1 | Drop genetic influences (A=0) | 0 | 24283.48 | 5491 | 13301.48 | 1 | 6.85e-19 | - | .74 | .41 | .41 | .29 | .64 |  |
| 2 | Drop shared environmental influences (C=o) | 0 | 24227.96 | 5491 | 13245.96 | 1 | 1.39e-06 | .84 | - | .54 | -.78 | .51 | .64 |  |
| 2 | Drop sex effect (b_sex_=0) | 0 | 24349.02 | 5491 | 13367.02 | 1 | 2.99e-33 | .75 | .38 | .54 | 1.11 | .32 | - |  |
| 3 | Drop phenotypic assortment and social homogamy (dp=0 and dy=0) | 0 | 24653.33 | 5492 | 13669.33 | 2 | 3.76^e^-98 | .73 | .43 | .53 | - | - | .64 |  |
| 5 | Drop phenotypic assortment (dp=0) | 0 | 24271.94 | 5491 | 13289.94 | *1* | 2.37^e^-16 | .55 | .61 | .56 | 1.09 | - | .65 |  |
| 6 | Drop social homogamy (dy=0) | 0 | 24227.96 | 5491 | 13245.96 | *1* | 1.39^e^-06 | .84 | 6.03 e-06 | .54 | - | .51 | .64 |  |
| The Netherlands | | | | | | | | | | | | | | |
| 0 | **Full model: A; C; E; dy, dp** | **-** | **22614.50** | **5618** | **11378.50** |  |  | **.81** | **.36** | **.46** | **1.18** | **.30** | **.20** |  |
| 1 | Drop genetic influences (A=0) | 0 | 22729.12 | 5619 | 11491.12 | *1* | 9.54e-27 | - | .81 | .59 | .36 | .22 | .20 |  |
| 2 | Drop social background influences (C=0) | 0 | 22637.99 | 5619 | 11399.99 | *1* | 1.26e-06 | .89 | - | .47 | 23.78 | .45 | .21 |  |
| 3 | Drop sex effect (b_sex_=0) | 0 | 22633.74 | 5619 | 11395.74 | *1* | 1.16e-05 | .81 | .36 | .46 | 1.23 | ..28 | - |  |
| 4 | Drop phenotypic assortment and social homogamy (dp=0 and dy=0) | 0 | 23011.23 | 5620 | 11771.23 | 2 | 7.12e-87 | .77 | .44 | .46 | - | - | .16 |  |
| 5 | Drop phenotypic assortment (dp=0) | 0 | 22649.69 | 5619 | 11411.69 | *1* | 3.00e-09 | .63 | .62 | .47 | 1.03 | - | .19 |  |
| 6 | Drop social homogamy (dy=0) | 0 | 22637.99 | 5619 | 11399.99 | *1* | 1.26e-06 | .89 | .31 | .47 | - | .45 | .21 |  |
