# Supplementary material for: The mechanism of assortative mating for educational attainment: a study of Finnish and Dutch twins and their spouses
Source: Front Genet. 2023 Jun 14;14:1150697. doi: 10.3389/fgene.2023.1150697 (PMC10311485; doi:10.3389/fgene.2023.1150697)
Supplement: Supplementary file 2 [file DataSheet3.docx]

Supplementary Material

The mechanism of assortative mating for educational attainment: a study of Finnish and Dutch twins and their spouses

Bodine Gonggrijp*, K. Silventoinen, C.V. Dolan, D. Boomsmaa, J. Kaprio & G. Willemsen.

*** Correspondence:** Corresponding Author: b.m.a.gonggrijp@vu.nl

# Supplementary Figures and Tables 2

| Supplementary Table 2. *Correlation matrix between twins and spouses.* | | | | | | | | | | | | | |
| --- | --- | --- | --- | --- | --- | --- | --- | --- | --- | --- | --- | --- | --- |
|  | Finland | | | | |  |  | The Netherlands | | | | |  |
|  | All twins | MZm | MZf | DZm | DZf | Dos |  | All twins | MZm | MZf | DZm | DZf | Dos |
| ɾ_tw1_– ɾ_tw2_ | .53^**^ | .75^**^ | .68^**^ | .52^**^ | .54^**^ | .36^**^ |  | .60^**^ | .72^**^ | .74^**^ | .41^**^ | .44** | .47^**^ |
|  | (.50 - .57) | (.68 - .80) | (.61 - .73) | (.42 - .61) | (.45 - .62) | (.28 - .43) |  | (.57 - .64) | (.64 - .79) | (.70 - .78) | (.21 - 57) | (.32 - .54) | (.35 - .58) |
| ɾ_tw_– ɾ_sp_ | .48^**^ | .45^**^ | .46^**^ | .50^**^ | .53^**^ | .50^**^ |  | .45^**^ | .46^**^ | .44^**^ | .45^**^ | .44** | .50^**^ |
|  | (.43 - .51) | (.32 - .56) | (.37 - .55) | (.39 - .59) | (.44 - .62) | (.43 - .56) |  | (.41 - .49) | (.34 - .56) | (.37 - .50) | (.29 - .59) | (.35 - .52) | (.42 - .58) |
| ɾ_cotw_– ɾ_sp_ | .34^**^ | .36^**^ | .42^**^ | .38^**^ | .46^**^ | .25^**^ |  | .37^**^ | .39^**^ | .35^*^ | .35^*^ | .38** | .38^**^ |
|  | (.29 - .39) | (.21 - .49) | (.31 - .51) | (.24 - .51) | (.35 - .56) | (.16 - .34) |  | (.32 - .42) | (.24 - .53) | (.12 - .54) | (.19 - .54) | (.30 - .45) | (.25 - .50) |
| ɾ_sp1_–ɾ_sp2_ | .30^**^ | .10 | .39^**^ | .45^*^ | .45^**^ | .13 |  | .33^**^ | .65^**^ | .30^**^ | .46 | .42** | .12 |
|  | (.20 - .39) | (-.18 - .37) | (.20 - .56) | (.19 - .65) | (.23 - .62) | (-.06 - .31) |  | (.24 - .42) | (.65 - .82) | (.15 - .43) | (-.03 - .77) | (.23 - .58) | (-.14 - .37) |
| ^*^  Correlations are significant at the 0.05 level (2-tailed).  ^**^ Correlations are significant at the 0.01 level (2-tailed). | | | | | | | | | | | | | |
