# Supplementary material for: The mechanism of assortative mating for educational attainment: a study of Finnish and Dutch twins and their spouses
Source: Front Genet. 2023 Jun 14;14:1150697. doi: 10.3389/fgene.2023.1150697 (PMC10311485; doi:10.3389/fgene.2023.1150697)
Supplement: Supplementary file 3 [file DataSheet5.docx]

Supplementary Material

The mechanism of assortative mating for educational attainment: a study of Finnish and Dutch twins and their spouses

Bodine Gonggrijp*, K. Silventoinen, C.V. Dolan, D. Boomsmaa, J. Kaprio & G. Willemsen.

*** Correspondence:** Corresponding Author: b.m.a.gonggrijp@vu.nl

# Supplementary Figures and Tables

| Supplementary Table 4. *Parameter estimates with 95% confidence intervals (95% CI) for full model of educational attainment.* | | | | | | |
| --- | --- | --- | --- | --- | --- | --- |
|  | Finland | | | The Netherlands | | |
|  |  | 95% CI | |  | 95% CI | |
|  | Parameter estimate | LL | UL | Parameter estimate | LL | UL |
| h | .74 | .66 | .81 | .81 | .72 | .88 |
| c | .39 | .21 | .49 | .36 | .15 | .51 |
| e | .54 | .51 | .57 | .46 | .44 | .49 |
| d_y_ | 1.03 | .70 | 1.69 | 1.18 | .77 | - |
| d_p_ | .35 | .27 | .43 | .30 | .20 | .40 |
| b_sex_ | .64 | .54 | .75 | .20 | .11 | .29 |
| h = square root of additive genetic factors; e = square root of unique environmental factors; c = square root of shared environmental factors; d_y_ = social homogamy; d_p_ = phenotypic assortment and b_sex_ = sex effect. | | | | | | |
