# Supplementary material for: The mechanism of assortative mating for educational attainment: a study of Finnish and Dutch twins and their spouses
Source: Front Genet. 2023 Jun 14;14:1150697. doi: 10.3389/fgene.2023.1150697 (PMC10311485; doi:10.3389/fgene.2023.1150697)
Supplement: Supplementary file 4 [file DataSheet2.docx]

Supplementary Material

The mechanism of assortative mating for educational attainment: a study of Finnish and Dutch twins and their spouses

Bodine Gonggrijp*, K. Silventoinen, C.V. Dolan, D. Boomsmaa, J. Kaprio & G. Willemsen.

*** Correspondence:** Corresponding Author: b.m.a.gonggrijp@vu.nl

# Supplementary Figures and Tables

| Supplementary Table 1. *Prevalence of completed educational level in Finland and the Netherlands* | | | |
| --- | --- | --- | --- |
| **Finland** | | **The Netherlands** | |
| **Educational level** | **% (N)** | **Educational level** | **% (N)** |
| Junior high school | 2.8 (161) | Primary education | 1.5 (79) |
| Vocational School | 21.6 (1209) | Lower vocational schooling / Lower secondary schooling (general) | 11.6 (619) |
| College level/ Senior High School | 21.6 (1207) | Intermediate vocational schooling / higher secondary schooling | 35.2 (1871) |
| University of Applied Sciences | 26.2 (1468) | Higher vocational schooling | 28.3 (1507) |
| College or University | 27.8 (1555) | University | 23.4 (1245) |
| Total | 5600 |  | 5321 |
