# Supplementary material for: The mechanism of assortative mating for educational attainment: a study of Finnish and Dutch twins and their spouses
Source: Front Genet. 2023 Jun 14;14:1150697. doi: 10.3389/fgene.2023.1150697 (PMC10311485; doi:10.3389/fgene.2023.1150697)
Supplement: Supplementary file 5 [file DataSheet1.docx]

Supplementary Material

The mechanisms of assortative mating for educational attainment: Insights from two European twin-spouse cohorts

Bodine Gonggrijp*, K. Silventoinen, C.V. Dolan, D. Boomsmaa, J. Kaprio & G. Willemsen.

*** Correspondence:** Corresponding Author: b.m.a.gonggrijp@vu.nl

# Supplementary Data

**Implementation of the different models of Reynolds et. al in OpenMx, provided with original script and power calculation.**

The models discussed in this document address similarity among spouses in the twin design. The sampling unit is a twin pair (t1, t2), plus the spouses (s1,s2): t1 - s1 - t2 - s2, i.e., 4 phenotype values per sampling unit. The models aim to resolve phenotypic assortment and social homogamy. Three models are considered; the Factor/Delta Path Homogamy model and the Delta/Delta Path Homogamy model both derived from Reynolds et al. (1996) and the model derived from Reynolds et al. 2006.

**Model 1: Factor/Delta Path Homogamy model**

**Figure 1.** Assortative mating predicted by the factor/delta path model of assortment. P = observed phenotype, A = additive genetic value; S = Social Homogamy environmental value; E = environmental deviations; h, s and e = path regressions of A, S and E; c = correlation among twin environment, Δp = selective association between phenotypes of spouses. This model is derived from Reynolds et al. 1996.


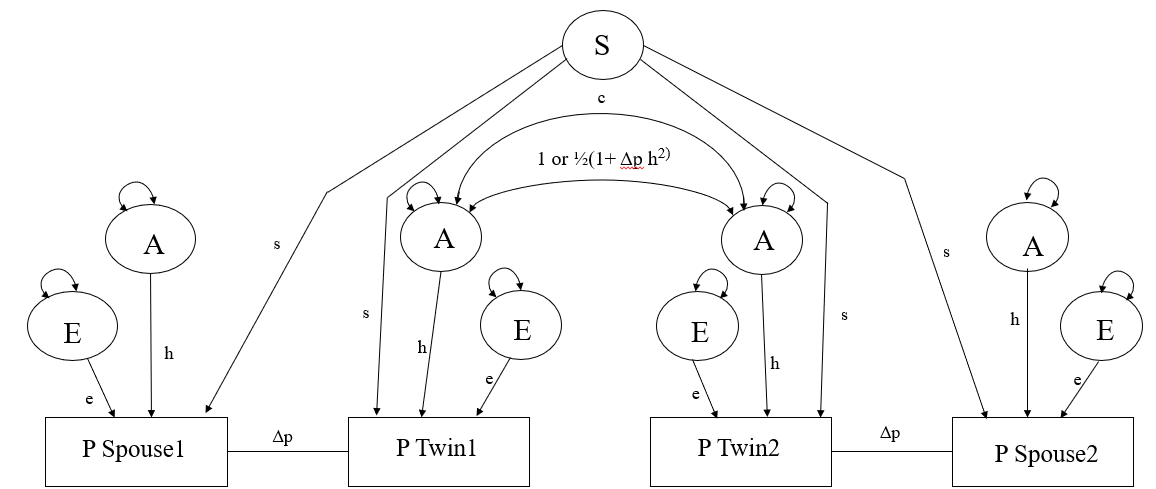


Social homogamy is modeled as an extrafamilial source of shared environment (S), in the presence of phenotypic assortment (Δp). The expected correlations are given in Table 1. If phenotypic assortment is the sole assortment process, the correlations are expected to follow a pattern of: spouse > cotwin-spouse > spouse1-spouse2. In the case of pure social homogamy, correlations are expected to be equal.

| **Table 1.** Expected correlations under the Factor/Delta Path Homogamy Model | | |
| --- | --- | --- |
|  | MZ | DZ |
| Twin | h^2^ + ce^2^ + s^2^ | ½h^2^(1 + Δ_p_ h^2^ ) + ce^2^ + s^2^ |
| Spouse | Δ_p_ + s^2^ | Δ_p_ + s^2^ |
| Cotwin - spouse | Δ_p_*r*_mz_ + s^2^ | Δ_p_*r_dz_* + s^2^ |
| Spouse1 - spouse2 | Δ_p_^2^*r*_mz_ + s^2^ + 2Δp s^2^ | Δ_p_^2^*r*_dz_ + s^2^ + 2Δp s^2^ |

Appendix 1 contains the OpenMx script for the Factor/Delta Path homogamy model. This implements the expected correlations as given in Table I. The script consists of 1) R code data simulation part; 2) R code OpenMx script). Running the script will give the following results.

> summary(r1DF)

Summary of DFM_RBP

free parameters:

name matrix row col Estimate

1 path_h DFM.h 1 1 0.6324550

2 path_e DFM.e 1 1 0.6324561

3 path_s DFM.s 1 1 0.4472135

4 cor_c DFM.c 1 1 0.2500009

5 ph_am DFM.dm 1 1 0.3000000

6 phm DFMMZ.mzphme 1 T1 5.0000000

7 phs DFMMZ.mzphsd 1 1 1.9999998

Note: these are the result specified in the simulation part of the script (i.e., e2=h2=.63245^2 = .4, e2=.44721^2=.2, c=.25, δm (ph_am)=.3, mean and variance are 5 and 4 (std = 2)).

Comparison to the saturated model:

base comparison ep minus2LL df AIC diffLL diffdf p

1 Saturated DFM_RBP <NA> 28 7877.916 1972 3933.916 NA NA NA

2 Saturated DFM_RBP DFM_RBP 7 7877.916 1994 3889.916 -1.215296e-06 22 1

Test of phenotypic AM (parameter dm in Table 1 i.e., ph_am here):

base comparison ep minus2LL df AIC diffLL diffdf p

1 DFM_RBP <NA> 7 7877.916 1994 3889.916 NA NA NA

2 DFM_RBP DFM_RBP 6 7911.005 1995 3921.005 33.0893 1 8.802182e-09

Test of soc homogamy (parameter s in Table 1, i.e., s here)

1 DFM_RBP <NA> 7 7877.916 1994 3889.916 NA NA NA

2 DFM_RBP DFM_RBP 6 7912.075 1995 3922.075 34.15904 1 5.078665e-09

Test of both (implying random mating)

base comparison ep minus2LL df AIC diffLL diffdf p

1 DFM_RBP <NA> 7 7877.916 1994 3889.916 NA NA NA

2 DFM_RBP DFM_RBP 5 8199.757 1996 4207.757 321.8411 2 1.297447e-70

The power calculation is conducted as follows (e.g., test of δm=0):

#

getchipow=function(alpha,df,Tval) {

ca=qchisq(alpha,df,ncp=0,lower.tail=F)

# critical value given alpha

power=pchisq(ca,df,ncp=Tval,lower.tail=F)

power

}

print(getchipow(.05, 1, 33.0893))

The power to detect the presence of phenotypic assortment given alpha=0.05 is large: .999.

**Summary of model 2: Delta/Delta Path Homogamy model (Reynolds et al., 1996).**


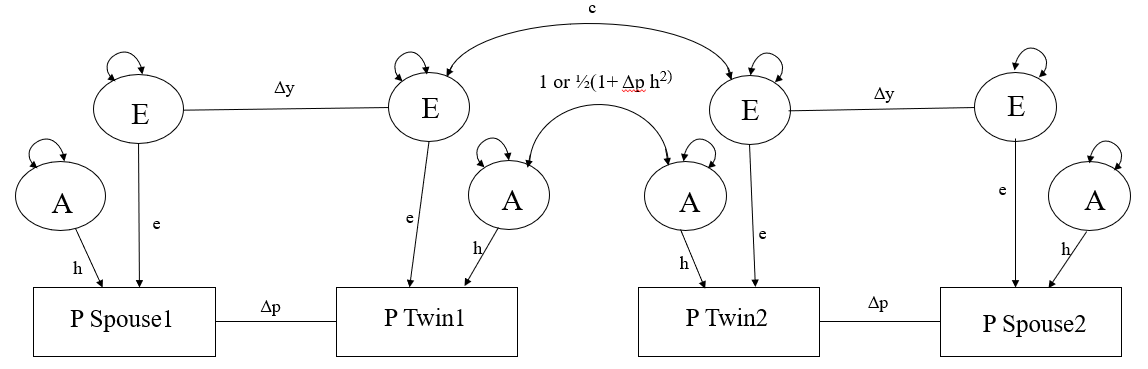


**Figure 2.** Assortative mating predicted by the delta/delta path model of assortment. P = observed phenotype, A = additive genetic value; E = environmental deviations; h and e = path regressions of A and E; c = correlation among twin environment; Δp = selective association between phenotypes of spouses; Δy = selective association between environment values of spouses. This model is derived from Reynolds et al. 1996.

Social homogamy is represented by the delta path Δy, connecting the environmental factors E, so here (in contrast to Figure 2), social homogamy is an indirect effect on the phenotypes via the factors E. This represents a matching with respect to E, a source of covariance not a source of variance (in contrast to the S in model 1, which is both). Table 2 shows the expected correlations under the delta/delta path homogamy model.

| **Table 2.** Expected correlations under the Delta/Delta Path Homogamy Model | | |
| --- | --- | --- |
|  | MZ | DZ |
| Twin | h^2^ + ce^2^ | ½h^2^(1 + Δ_p_ h^2^ ) + ce^2^ |
| Spouse | Δ_p_ + Δ_y_e^2^ | Δ_p_ + Δ_y_e^2^ |
| Cotwin - spouse | Δ_p_*r*_mz_ + Δ_y_ce^2^ | Δ_p_*r*_dz_ + Δ_y_ce^2^ |
| Spouse1 - spouse2 | Δ_p_^2^*r*_mz_ 2Δ_p_Δ_y_ce^2^ + Δ_y_ce^2^ | Δ_p_^2^*r*_dz_ 2Δ_p_Δ_y_ce^2^ + Δ_y_ce^2^ |

The appendix 2 contains the OpenMx script. This implements the expected correlations as given in Table II. The script consists of 1) R code data simulation part; 2) R code OpenMx script). Running the script from appendix 2 gives the following results.

Summary of DDM_RBP

free parameters:

name matrix row col Estimate

1 path_h DDM.h 1 1 0.7071061

2 path_e DDM.e 1 1 0.7071075

3 e_am DDM.ds 1 1 0.2000009

4 cor_c DDM.c 1 1 0.2500010

5 ph_am DDM.dm 1 1 0.1499994

6 phm DDMMZ.mzphme 1 T1 4.9999999

7 phs DDMMZ.mzphsd 1 1 1.9999998

Note: these are the result specified in the simulation part of the script (e2=h2=.7071^2 = .5, e2=.7071^2=.5, c=.25, δm (ph_am)=.15, δs (e_am) = .20, mean and variance are 5 and 4 (std = 2)).

Comparison to the saturated model:

base comparison ep minus2LL df AIC diffLL diffdf p

1 Saturated DDM_RBP <NA> 28 8216.473 1972 4272.473 NA NA NA

2 Saturated DDM_RBP DDM_RBP 7 8216.473 1994 4228.473 -8.491224e-08 22 1

Test of phenotypic AM (parameter dm in Table 1 i.e., ph_am here):

base comparison ep minus2LL df AIC diffLL diffdf p

1 DDM_RBP <NA> 7 8216.473 1994 4228.473 NA NA NA

2 DDM_RBP DDM_RBP 6 8217.525 1995 4227.525 1.051605 1 0.3051378

Test of soc homogamy (parameter s in Table 1, i.e., s here)

base comparison ep minus2LL df AIC diffLL diffdf p

1 DDM_RBP <NA> 7 8216.473 1994 4228.473 NA NA NA

2 DDM_RBP DDM_RBP 6 8217.858 1995 4227.858 1.384279 1 0.2393736

Test of both (implying random mating)

base comparison ep minus2LL df AIC diffLL diffdf p

1 DDM_RBP <NA> 7 8216.473 1994 4228.473 NA NA NA

2 DDM_RBP DDM_RBP 5 8282.396 1996 4290.396 65.9228 2 4.842234e-15

Parameter Δp and Δy are highly correlated, and thus very hard to resolve (see also Reynolds et al., 1996). The power to detect phenotypic assortment and social homogamy separately is low. But the power to detect both is >.9999 (alpha=0.05).

**Summary of model 3: Full model.**

**Figure 3.** Spousal selection based on phenotype and social background in twins reared together (TRT) or apart (TRA). P = observed phenotype, A = additive genetic value; C = Shared environmental factors E = unique environmental factors; S = social background environment; J = additional shared rearing environmental factors; h, c, e, s and j = path regressions of A, C, E, S and J, respectively; α = genetic similarity DZ twins (1/2(1 + Δp). Δp = selection based on phenotype; Δy = selection based on social background environment. This model is derived from Reynolds et al. 2006.


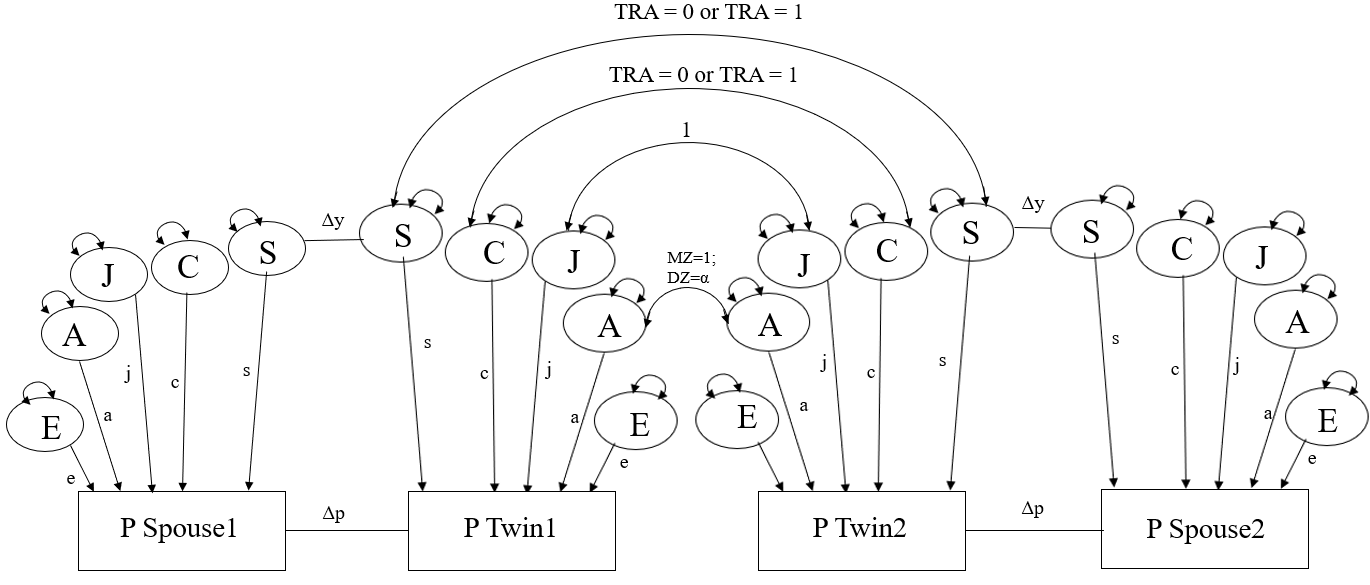


The model estimates four sources of environmental variance, namely: a shared environmental eﬀect that includes social background inﬂuences (S), a shared environmental eﬀect that accounts for any additional shared environmental eﬀects beyond social background (J), a correlated environmental eﬀect which increases the similarity of all siblings whether reared together or apart (C) and non-shared environmental inﬂuences (E). Phenotypic assortment is represented by a delta path (Δp), or direct associations, between the phenotypes of spouses. Social homogamy is represented by the delta path Δy, or direct associations, between the social background factors (S) that contribute to the traits in question. Table 3 shows the expected correlations under the model depicted in figure 3.

| **Table 3.** Univariate equations for the correlation among spouse pairs under the full model. | |
| --- | --- |
|  | Correlation |
| TMZT=rMZT | h2+s2+j2+c2 |
| TDZT=rDZT | 1/2h2(1+dPh)+s2+j2+c2 |
| TMZA=rMZA | h2+c2 |
| r_spouse_ | dP+dYsr_spouse_ |
| r_cotwin_ - r_spouse_ | dP+dYs |
| T = twin similarity MZ or DZ, either together (T) or apart (A) | |

**Applying the model of Reynolds et al. (2006) to MZ and DZ twins and their spouses.**

When applying the extended design of Reynolds et al (2006) to MZ and DZ twins and their spouses, we cannot estimate J, S and C effects, as these factors are all correlated 1. Thus the question is if we can use the extended model of Reynolds et al. (2006) to MZ and DZ twins and their spouses to estimate both S and C? The appendix 3 contains the OpenMx script. The script consists of 1) R code data simulation part; 2) R code OpenMx script). Running the script from appendix 2 gives the following results.

Parameters, where C and S and Δy are large:

h=sqrt(.40) # h^2 sqrt(h^2) = h # additive genetic effect of factor A

c=sqrt(.1)

s=sqrt(.40)

# h^2 + c^2 + s^2 < 1

e=sqrt(1-h^2-c^2-s^2)

dp=.20 # social homogamy effect E matching process

dy=.30 # phenotypic assortment Phenotypic matching

The true values are recovered without a problem:

estimated:

h e dy c s dp

0.632 0.316 0.300 0.316 0.632 0.200

true:

h e dy c s dp

0.632 0.316 0.300 0.316 0.632 0.200

Comparison to the saturated model:

base comparison ep minus2LL df AIC diffLL diffdf p

1 DDM_RBP <NA> 8 49708.42 19993 9722.424 NA NA NA

2 DDM_RBP DDM_RBP 7 49708.69 19994 9720.687 0.263724 1 0.6075732

We see that the power to detect C is very low.

Parameters, where C and S and δy are relatively low:

h=sqrt(.40) # h^2 sqrt(h^2) = h # additive genetic effect of factor A

c=sqrt(.35)

s=sqrt(.10)

# h^2 + c^2 + s^2 < 1

e=sqrt(1-h^2-c^2-s^2)

dp=.20 # social homogamy effect E matching process

dy=.05 # phenotypic assortment Phenotypic matching

Estimated

h e dy c s dp

0.632 0.387 0.041 0.571 0.352 0.200

True:

h e dy c s dp

0.632 0.387 0.050 0.592 0.316 0.200

In this case, the parameters dy, c and s are not recovered correctly. This is due to empirical under identification.

**Solution**

Drop either C or S from the model and proceed. This will make the Reynolds et al. (2006) model like the Delta/Delta Path Homogamy model from Reynolds et al. (1996), except in the Delta/Delta model social homogamy is with respect to the total environment (E+C), whereas in the adjusted 2006 model social homogamy is with respect to either C or S, which includes true C and factors such as SES.


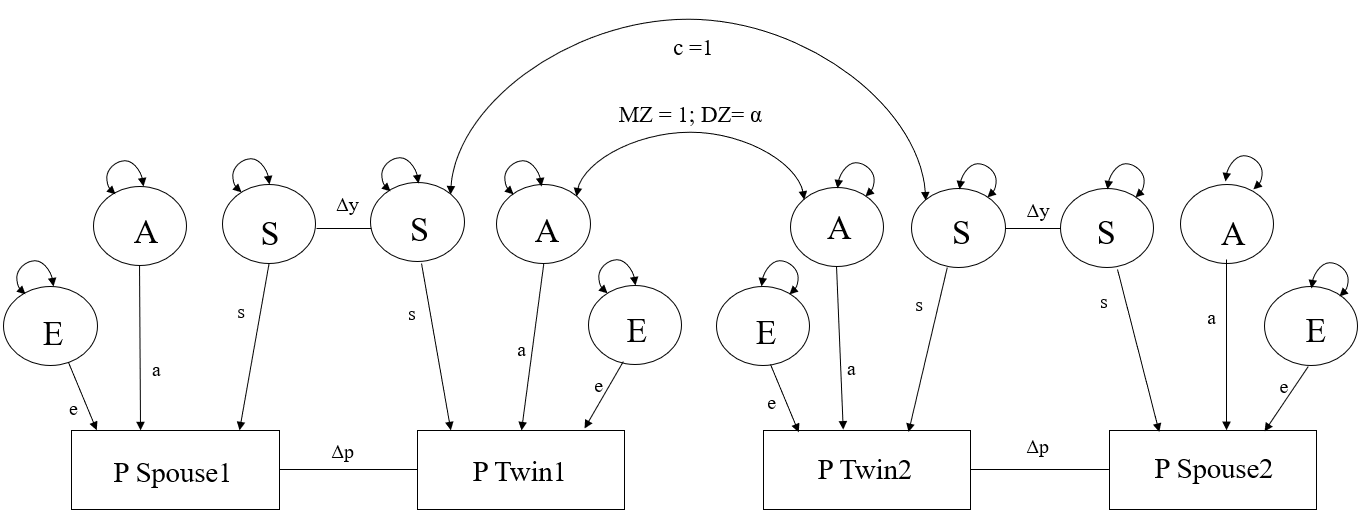


**Figure 4.** Proposed model of spouse selection on phenotype and social background, adjusted from full model in Reynolds et al. (2006). a = genetic path matrix; s = social background path matrix, which includes shared environmental influences (c); e= environmental path matrix; Δ_Y_ = social background delta path matrix; Δ_p_ = phenotype delta path matrix. The A factors are constrained to 1.0 for MZ pairs and α = DZ genetic similarity α = .5 (1 + .5Δ_p_ ) for DZ pairs and the C factors are constrained to 1 for both MZ and DZ pairs.

**Appendix 1: DF model (table 1 BRP, 1996). Annotated, data simulation and OpenMx script.**

# Factor/Delta Path Homogamy model

#

rm(list=ls(all=TRUE))

wr=TRUE # write data to external file j1dat?

# --------------------------------

library(OpenMx)

# simulation, we need a function in MASS.

library(MASS)

#

# ---------------------------- simulation specs.

# there are 7 parameters in this model

# h, e, s, c, dm (see RBP, 1996 Table 1 plus phenotypic mean and variance

exact=T # exact data simulation for power calc / script checking

Nmz=1000 # number of mz families (each 4 members t1-s1-t2-s2)

Ndz=1000 # number of dz families (each 4 members t1-s1-t2-s2)

# .4, .4, .2 .25 arbitrary values

h=sqrt(.4) # h^2 sqrt(h^2) = h # additive genetic effect of factor A

e=sqrt(.4) # e^2 sqrt(e^2) = e # environmental effect of factor E

s=sqrt(.2) # s^2 sqrt(s^2) = s # social homogamy effect of factor S contribution to correlation

c=.25 # correlation between E twin 1 and E twin 2 ... shared env effects

# NB: .4+.4+.2 = 1 ............... standardized model

h2=h^2

e2=e^2

s2=s^2

# h2+e2+s2 = 1 .................... standardized model

#

# delta_mu phenotypic assortment (copath / delta path)

# .3 arbitrary value

dm=.3 # phenotypic AM contribution to correlation

#

# the model is specified for correlation matrices but fitted to covariance matrices

# mean and variance of phenotypes

# arbitrary values

phvar=4 # phenotypic variance

phsd=sqrt(phvar); SD=diag(rep(phsd,4))

phmean=5 # phenotypic mean

#

# --------------- MZ expected correlations (Table 1)

# order of members: t1 s1 t2 s2

Rmz=matrix(0,4,4)

diag(Rmz) = 1

# MZ1 - MDZ2 (3,1)

rmz=Rmz[1,3]=Rmz[3,1]=h2+c*e2+s2

# MZ1 - spouse1(2,1) MZ2 - spouse2 (4,3)

Rmz[1,2]=Rmz[2,1]=dm+s2

Rmz[3,4]=Rmz[4,3]=dm+s2

# cotwin spouse, MZ1 - spouse2, MZ2 - spouse 1 (4,1) (3,2)

Rmz[4,1] = Rmz[1,4] = Rmz[3,2] = Rmz[2,3] = dm*rmz+s2

# spouse 1 spouse 2 (4,2)

Rmz[4,2]=Rmz[2,4]= dm^2*rmz + s2 + 2*dm*s2

# --------------------------------------------------

# --------------- DZ expected correlations (Table 1)

# order of members: t1 s1 t2 s2

Rdz=matrix(0,4,4)

diag(Rdz) = 1

# DZ1 - DZ2 (3,1)

rdz=Rdz[1,3]=Rdz[3,1]=.5*h2*(1+dm*h2) + c*e2 + s2

# DZ1 - spouse1(2,1) DZ2 - spouse2 (4,3)

Rdz[1,2]=Rdz[2,1]=dm+s2

Rdz[3,4]=Rdz[4,3]=dm+s2

# cotwin spouse, DZ1 - spouse2, DZ2 - spouse 1 (4,1) (3,2)

Rdz[4,1] = Rdz[1,4] = Rdz[3,2] = Rdz[2,3] = dm*rdz+s2

# spouse 1 spouse 2 (4,2)

Rdz[4,2]=Rdz[2,4]= dm^2*rdz + s2 + 2*dm*s2

# --------------------------------------------------

# from correlation matrices to covariance matrices

Smz=SD%*%Rmz%*%SD # covariance matrix

Mmz=rep(phmean,4) # means

Sdz=SD%*%Rdz%*%SD # covariance matrix

Mdz=rep(phmean,4) # means

# --------------------------- simulate data

cNmz=cNdz=1

# following only for exact data simulation

if (exact) {cNmz=(Nmz-1)/Nmz; cNdz=(Ndz-1)/Ndz}

datmz=mvrnorm(Nmz,mu=Mmz, Sigma=Smz/cNmz, emp=T)

datdz=mvrnorm(Ndz,mu=Mdz, Sigma=Sdz/cNdz, emp=T)

# write to external file?

if (wr) {

dat=matrix(2,Nmz+Ndz,5)

dat[1:Nmz,1]=1 # mz code 1

dat[1:Nmz,2:5]=datmz #

dat[(Nmz+1):(Nmz+Ndz),2:5]=datdz

write.table(dat,file="datj1",col.names=F, row.names=F)

}

#

# ---------------------------------------------- end simulation

colnames(datmz)=vnames=c('T1','S1','T2','S2')

colnames(datdz)=vnames=c('T1','S1','T2','S2')

datmz=as.data.frame(datmz) # mz dataframe

datdz=as.data.frame(datdz) # dz dataframe

# ------------------------------------------------ openmx spec

# ----------- openmx

# modelDF = table 1 implementation

#

# NB: starting values for h,e,s,c,dm (standardized matrices)

#

ModelDF = mxModel(model="DFM",

# parameters

mxMatrix(type="Full", nrow=1, ncol=1, free=TRUE, values=.5, label='path_h', name='h'), # add gen

mxMatrix(type="Full", nrow=1, ncol=1, free=TRUE, values=.5, label='path_e', name='e'), # env

mxMatrix(type="Full", nrow=1, ncol=1, free=TRUE, values=.5, label='path_s', name='s'), # social homog

mxMatrix(type="Full", nrow=1, ncol=1, free=TRUE, values=.2, label='cor_c', name='c'), # r(E1,E2)

mxMatrix(type="Full", nrow=1, ncol=1, free=TRUE, values=.2, label='ph_am', name='dm'), # delta_mu

mxMatrix(type='Full', nrow=1, ncol=1, free=FALSE, values=1, label='o1', name='O1'),

#

# mxMatrix(type='Full',nrow=1, ncol=1, free=TRUE, label='vph', name='vPh'),

#

# derived

mxAlgebra(expression=e %*% e, name = 'e2'),

mxAlgebra(expression=h %*% h, name = 'h2'),

mxAlgebra(expression=s %*% s, name = 's2'),

mxAlgebra(expression=dm %*% dm, name = 'dm2'),

#

# Table 1 RBP 1996

#

mxAlgebra(expression=e%*%e+h%*%h+s%*%s,name='vph'),

mxConstraint(vph==O1, name='c_stdvar'), # standardized

# correlation elements MZ - RBP 1996 table 1

mxAlgebra(expression=h2+c*e2+s2, name='rmzmz'),

mxAlgebra(expression=dm+s2, name='rmzsp'),

mxAlgebra(expression=dm*(h2+c*e2+s2)+s2, name='rmzctsp'),

mxAlgebra(expression=dm2*(h2+c*e2+s2)+s2+2*dm*s2, name='rmzspsp'),

# correlation elements DZ - RBP 1996 table 1

mxAlgebra(expression=.5*h2*(1+dm*h2)+c*e2+s2, name='rdzdz'),

mxAlgebra(expression=dm+s2, name='rdzsp'), # same as DZ

mxAlgebra(expression=dm*(.5*h2*(1+dm*h2)+c*e2+s2)+s2, name='rdzctsp'),

mxAlgebra(expression=dm2*(.5*h2*(1+dm*h2)+c*e2+s2)+s2+2*dm*s2, name='rdzspsp')

)

#

# starting values phm and phs (from cor to cov)

#

ModelDF_MZ = mxModel(model="DFMMZ",

mxData(observed=datmz, type='raw'),

mxMatrix(type="Full", nrow=1, ncol=4, free=TRUE, values=5, label=c('phm','phm','phm','phm'), name='mzphme'),

mxMatrix(type="Diag", nrow=4, ncol=4, free=TRUE, values=2, label=c('phs','phs','phs','phs'), name='mzphsd'),

mxAlgebra(expression=rbind(

cbind(1, DFM.rmzsp, DFM.rmzmz, DFM.rmzctsp),

cbind(DFM.rmzsp, 1, DFM.rmzctsp, DFM.rmzspsp),

cbind(DFM.rmzmz, DFM.rmzctsp, 1, DFM.rmzsp),

cbind(DFM.rmzctsp,DFM.rmzspsp, DFM.rmzsp, 1)),

name='mzcor'),

mxAlgebra(expression=mzphsd%*%mzcor%*%mzphsd, name='mzcov'),

# mxAlgebra(expression=DFM.vph%x%mzcor, name='mzcov'),

mxExpectationNormal(covariance='mzcov', means='mzphme', vnames),

mxFitFunctionML()

)

#

# starting values phm and phs should equal those in DFMDZ

#

ModelDF_DZ = mxModel(model="DFMDZ",

mxData(observed=datdz, type='raw'),

mxMatrix(type="Full", nrow=1, ncol=4, free=TRUE, values=5, label=c('phm','phm','phm','phm'), name='dzphme'),

mxMatrix(type="Diag", nrow=4, ncol=4, free=TRUE, values=2, label=c('phs','phs','phs','phs'), name='dzphsd'),

mxAlgebra(expression=rbind(

cbind(1, DFM.rdzsp, DFM.rdzdz, DFM.rdzctsp),

cbind(DFM.rdzsp, 1, DFM.rdzctsp, DFM.rdzspsp),

cbind(DFM.rdzdz, DFM.rdzctsp, 1, DFM.rdzsp),

cbind(DFM.rdzctsp,DFM.rdzspsp, DFM.rdzsp, 1)),

name='dzcor'),

mxAlgebra(expression=dzphsd%*%dzcor%*%dzphsd, name='dzcov'),

# mxAlgebra(expression=DFM.vph%x%dzcor, name='dzcov'),

mxExpectationNormal(covariance='dzcov', means='dzphme', vnames),

mxFitFunctionML()

)

# assemble

#

ModelDF_RBP = mxModel(model="DFM_RBP",ModelDF,ModelDF_MZ,ModelDF_DZ,

# old

# mxAlgebra(expression=DFMMZ.objective + DFMDZ.objective, name='DFfit'),

# mxFitFunctionAlgebra('DFfit')

# new way to do this

mxFitFunctionMultigroup( c("DFMMZ","DFMDZ") )

)

# run 7 parameter model full DF model

#

r1DF = mxRun(ModelDF_RBP)

Ir1DF=round(cov2cor(vcov(r1DF)),3)

#

# fit saturated model ................

r2Sat = mxRefModels(r1DF,run=TRUE)

mxCompare(r2Sat,r1DF) # if exact = T the likelihood ratio should be zero.

#

# test dm - phenotypic AM

#

ModelDF_RBP1=omxSetParameters(ModelDF_RBP, labels=c('ph_am'), values=0, free=FALSE)

r1DFdm = mxRun(ModelDF_RBP1)

mxCompare(r1DF,r1DFdm)

#

# test s soc homogamy

#

ModelDF_RBP2=omxSetParameters(ModelDF_RBP, labels=c('path_s'), values=0, free=FALSE)

r1DFs = mxRun(ModelDF_RBP2)

mxCompare(r1DF,r1DFs)

# test both - meaning random mating

#

ModelDF_RBP3=omxSetParameters(ModelDF_RBP, labels=c('ph_am','path_s'), values=0, free=FALSE)

r1DFsdm = mxRun(ModelDF_RBP3)

mxCompare(r1DF,r1DFsdm)

**Appendix 2: DD model (table 2 BRP, 1996). Annotated, data simulation and OpenMx script**

# Delta/Delta Path model

#

rm(list=ls(all=TRUE))

#

wr=TRUE # if true write data to external file datj2

# --------------------------------

library(OpenMx)

# simulation, we need a function in MASS.

library(MASS)

#

# ---------------------------- simulation specs.

# there are 7 parameters in this model

# h, e, c, dm, ds (see RBP, 1996 Table 2 plus phenotypic mean and variance

exact=T # exact data simulation for power calc / script checking

Nmz=250 # number of mz families (each 4 members t1-s1-t2-s2)

Ndz=250 # number of dz families (each 4 members t1-s1-t2-s2)

# . arbitrary values

h=sqrt(.5) # h^2 sqrt(h^2) = h # additive genetic effect of factor A

e=sqrt(.5) # e^2 sqrt(e^2) = e # environmental effect of factor E

ds=.2 # social homogamy effect E matching process

dm=.15 # phenotypic assortment Phenotypic matching

c=.25 # correlation between E twin 1 and E twin 2 ... shared env effects

# NB: .5+.5 = 1 ............... standardized model

h2=h^2

e2=e^2

# h2+e2 = 1 .................... standardized model

#

#

# the model is specified for correlation matrices but fitted to covariance matrices

# mean and variance of phenotypes

# arbitrary values

phvar=4 # phenotypic variance

phsd=sqrt(phvar); SD=diag(rep(phsd,4))

phmean=5 # phenotypic mean

#

# --------------- MZ expected correlations (Table 1)

# order of members: t1 s1 t2 s2

Rmz=matrix(0,4,4)

diag(Rmz) = 1

# MZ1 - MDZ2 (3,1)

rmz=Rmz[1,3]=Rmz[3,1]=h2+c*e2

# MZ1 - spouse1(2,1) MZ2 - spouse2 (4,3)

Rmz[1,2]=Rmz[2,1]=dm+ds*e2

Rmz[3,4]=Rmz[4,3]=dm+ds*e2

# cotwin spouse, MZ1 - spouse2, MZ2 - spouse 1 (4,1) (3,2)

Rmz[4,1] = Rmz[1,4] = Rmz[3,2] = Rmz[2,3] = dm*rmz+ds*c*e2

# spouse 1 spouse 2 (4,2)

Rmz[4,2]=Rmz[2,4]= dm^2*rmz + 2*dm*ds*c*e2 + ds^2*c*e2

# --------------------------------------------------

# --------------- DZ expected correlations (Table 1)

# order of members: t1 s1 t2 s2

Rdz=matrix(0,4,4)

diag(Rdz) = 1

# DZ1 - DZ2 (3,1)

rdz=Rdz[1,3]=Rdz[3,1]=.5*h2*(1+dm*h2) + c*e2

# DZ1 - spouse1(2,1) DZ2 - spouse2 (4,3)

Rdz[1,2]=Rdz[2,1]=dm+ds*e2

Rdz[3,4]=Rdz[4,3]=dm+ds*e2

# cotwin spouse, DZ1 - spouse2, DZ2 - spouse 1 (4,1) (3,2)

Rdz[4,1] = Rdz[1,4] = Rdz[3,2] = Rdz[2,3] = dm*rdz+ds*c*e2

# spouse 1 spouse 2 (4,2)

Rdz[4,2]=Rdz[2,4]= dm^2*rdz + 2*dm*ds*c*e2 +ds^2*c*e2

# --------------------------------------------------

# from correlation matrices to covariance matrices

Smz=SD%*%Rmz%*%SD # covariance matrix

Mmz=rep(phmean,4) # means

Sdz=SD%*%Rdz%*%SD # covariance matrix

Mdz=rep(phmean,4) # means

# --------------------------- simulate data

cNmz=cNdz=1

# following only for exact data simulation

if (exact) {cNmz=(Nmz-1)/Nmz; cNdz=(Ndz-1)/Ndz}

datmz=mvrnorm(Nmz,mu=Mmz, Sigma=Smz/cNmz, emp=T)

datdz=mvrnorm(Ndz,mu=Mdz, Sigma=Sdz/cNdz, emp=T)

#

**# write to external file?**

if (wr) {

dat=matrix(2,Nmz+Ndz,5)

dat[1:Nmz,1]=1 # mz code 1

dat[1:Nmz,2:5]=datmz #

dat[(Nmz+1):(Nmz+Ndz),2:5]=datdz

write.table(dat,file="datj2",col.names=F, row.names=F)

}

#

# ---------------------------------------------- end simulation

**colnames(datmz)=vnames=c('T1','S1','T2','S2')**

**colnames(datdz)=vnames=c('T1','S1','T2','S2')**

**datmz=as.data.frame(datmz) # mz dataframe**

**datdz=as.data.frame(datdz) # dz dataframe**

# ------------------------------------------------ openmx spec

# modelDD = table 2 implementation

#

# **NB: starting values for h,e,c,dm, ds (standardized matrices)**

ModelDD = mxModel(model="DDM",

# parameters

mxMatrix(type="Full", nrow=1, ncol=1, free=TRUE, values=.5, label='path_h', name='h'), # add gen

mxMatrix(type="Full", nrow=1, ncol=1, free=TRUE, values=.5, label='path_e', name='e'), # env

mxMatrix(type="Full", nrow=1, ncol=1, free=TRUE, values=.1, label='e_am', name='ds'), # social homog

mxMatrix(type="Full", nrow=1, ncol=1, free=TRUE, values=.2, label='cor_c', name='c'), # r(E1,E2)

mxMatrix(type="Full", nrow=1, ncol=1, free=TRUE, values=.2, label='ph_am', name='dm'), # delta_mu

mxMatrix(type='Full', nrow=1, ncol=1, free=FALSE, values=1, label='o1', name='O1'),

#

# mxMatrix(type='Full',nrow=1, ncol=1, free=TRUE, label='vph', name='vPh'),

#

# derived

mxAlgebra(expression=e %*% e, name = 'e2'),

mxAlgebra(expression=h %*% h, name = 'h2'),

mxAlgebra(expression=dm %*% dm, name = 'dm2'),

mxAlgebra(expression=ds %*% ds, name = 'ds2'),

#

#

# Table 1 RBP 1996

#

mxAlgebra(expression=e%*%e+h%*%h,name='vph'),

mxConstraint(vph==O1, name='c_stdvar'), # standardized

# correlation elements MZ - RBP 1996 table 1

mxAlgebra(expression=h2+c*e2, name='rmzmz'),

mxAlgebra(expression=dm+ds*e2, name='rmzsp'),

mxAlgebra(expression=dm*(h2+c*e2)+ds*c*e2, name='rmzctsp'),

mxAlgebra(expression=dm2*(h2+c*e2)+2*dm*ds*c*e2+ds2*c*e2, name='rmzspsp'),

# correlation elements DZ - RBP 1996 table 1

mxAlgebra(expression=.5*h2*(1+dm*h2)+c*e2, name='rdzdz'),

mxAlgebra(expression=dm+ds*e2, name='rdzsp'), # same as DZ

mxAlgebra(expression=dm*(.5*h2*(1+dm*h2)+c*e2)+ds*c*e2, name='rdzctsp'),

mxAlgebra(expression=dm2*(.5*h2*(1+dm*h2)+c*e2)+2*dm*ds*c*e2 + ds2*c*e2, name='rdzspsp')

)

#

# starting values phm and phs (from cor to cov)

#

ModelDD_MZ = mxModel(model="DDMMZ",

mxData(observed=datmz, type='raw'),

mxMatrix(type="Full", nrow=1, ncol=4, free=TRUE, values=5, label=c('phm','phm','phm','phm'), name='mzphme'),

mxMatrix(type="Diag", nrow=4, ncol=4, free=TRUE, values=2, label=c('phs','phs','phs','phs'), name='mzphsd'),

mxAlgebra(expression=rbind(

cbind(1, DDM.rmzsp, DDM.rmzmz, DDM.rmzctsp),

cbind(DDM.rmzsp, 1, DDM.rmzctsp, DDM.rmzspsp),

cbind(DDM.rmzmz, DDM.rmzctsp, 1, DDM.rmzsp),

cbind(DDM.rmzctsp,DDM.rmzspsp, DDM.rmzsp, 1)),

name='mzcor'),

mxAlgebra(expression=mzphsd%*%mzcor%*%mzphsd, name='mzcov'),

# mxAlgebra(expression=DFM.vph%x%mzcor, name='mzcov'),

mxExpectationNormal(covariance='mzcov', means='mzphme', vnames),

mxFitFunctionML()

)

#

# starting values phm and phs should equal those in DFMDZ

#

ModelDD_DZ = mxModel(model="DDMDZ",

mxData(observed=datdz, type='raw'),

mxMatrix(type="Full", nrow=1, ncol=4, free=TRUE, values=5, label=c('phm','phm','phm','phm'), name='dzphme'),

mxMatrix(type="Diag", nrow=4, ncol=4, free=TRUE, values=2, label=c('phs','phs','phs','phs'), name='dzphsd'),

mxAlgebra(expression=rbind(

cbind(1, DDM.rdzsp, DDM.rdzdz, DDM.rdzctsp),

cbind(DDM.rdzsp, 1, DDM.rdzctsp, DDM.rdzspsp),

cbind(DDM.rdzdz, DDM.rdzctsp, 1, DDM.rdzsp),

cbind(DDM.rdzctsp,DDM.rdzspsp, DDM.rdzsp, 1)),

name='dzcor'),

mxAlgebra(expression=dzphsd%*%dzcor%*%dzphsd, name='dzcov'),

# mxAlgebra(expression=DFM.vph%x%dzcor, name='dzcov'),

mxExpectationNormal(covariance='dzcov', means='dzphme', vnames),

mxFitFunctionML()

)

#

# assemble

#

ModelDD_RBP = mxModel(model="DDM_RBP",ModelDD,ModelDD_MZ,ModelDD_DZ,

# old

# mxAlgebra(expression=DFMMZ.objective + DFMDZ.objective, name='DFfit'),

# mxFitFunctionAlgebra('DFfit')

# new way to do this

mxFitFunctionMultigroup( c("DDMMZ","DDMDZ") )

)

# run 7 parameter model full DD model

#

r1DD = mxRun(ModelDD_RBP)

Ir1DD=round(cov2cor(vcov(r1DD)),3)

#

# fit saturated model ................

r2Sat = mxRefModels(r1DD,run=TRUE)

mxCompare(r2Sat,r1DD) # if exact = T the likelihood ratio should be zero.

#

# test dm - phenotypic AM

#

ModelDD_RBP1=omxSetParameters(ModelDD_RBP, labels=c('ph_am'), values=0, free=FALSE)

r1DDdm = mxRun(ModelDD_RBP1)

mxCompare(r1DD,r1DDdm)

#

# test ds soc homogamy e matching

ModelDD_RBP2=omxSetParameters(ModelDD_RBP, labels=c('e_am'), values=0, free=FALSE)

r1DDs = mxRun(ModelDD_RBP2)

mxCompare(r1DD,r1DDs)

#

# test both - meaning random mating

ModelDD_RBP3=omxSetParameters(ModelDD_RBP, labels=c('ph_am','e_am'), values=0, free=FALSE)

r1DDsdm = mxRun(ModelDD_RBP3)

mxCompare(r1DD,r1DDsdm)

#

**Appendix 3: DD model BRP, 2006. Annotated, data simulation and OpenMx script**

# Delta/Delta Path model

# simplified version of RBP 2006 drop J as requested, retain S and C

# model correlations

#TMZ = rMZ = h2+s2+c2

#TDZ = rDZ = .5*h2*(1+dp*h2) + c2 + s2

#rSPOUSES = dp +s2*dy

#rCotwin-Spouse (MZs) = TMZ*dp + s2*dy

#rCotwin-Spouse (DZs) = TDZ*dp + s2*dy

#rSpouse1-Spouse2 (MZ) = TMZ*dp2 + s2*dy2 + 2*s2*dy*dp

#rSpouse1-Spouse2 (DZ) = TDZ*dp2 + s2*dy2 + 2*s2*dy*dp

#

rm(list=ls(all=TRUE))

#

wr=TRUE # if true write data to external file datj3

# --------------------------------

library(OpenMx)

# simulation, we need a function in MASS.

library(MASS)

#

# ---------------------------- simulation specs.

# there are 7 parameters in this model

# h, e, c, dm, ds (see RBP, 1996 Table 2 plus phenotypic mean and variance

exact=TRUE # exact data simulation for power calc / script checking

Nmz=2500 # number of mz families (each 4 members t1-s1-t2-s2)

Ndz=2500 # number of dz families (each 4 members t1-s1-t2-s2)

# . arbitrary values

h=sqrt(.40) # h^2 sqrt(h^2) = h # additive genetic effect of factor A

c=sqrt(.35)

s=sqrt(.10)

# h^2 + c^2 + s^2 < 1

e=sqrt(1-h^2-c^2-s^2)

dp=.20 # social homogamy effect E matching process

dy=.05 # phenotypic assortment Phenotypic matching

# NB: .5+.5 = 1 ............... standardized model

h2=h^2

c2=c^2

s2=s^2

e2=e^2

dp2=dp^2

dy2=dy^2

#

#

# the model is specified for correlation matrices but fitted to covariance matrices

# mean and variance of phenotypes

# arbitrary values

phvar=1 # phenotypic variance

phsd=sqrt(phvar); SD=diag(rep(phsd,4))

phmean=0 # phenotypic mean

#

#

#TMZ = rMZ = h2+s2+c2

#TDZ = rDZ = .5*h2*(1+dp*h2) + c2 + s2

#rSPOUSES = dp +s2*dy

#rCotwin-Spouse (MZs) = TMZ*dp + s2*dy

#rCotwin-Spouse (DZs) = TDZ*dp + s2*dy

#rSpouse1-Spouse2 (MZ) = TMZ*dp2 + s2*dy2 + 2*s2*dy*dp

#rSpouse1-Spouse2 (DZ) = TDZ*dp2 + s2*dy2 + 2*s2*dy*dp

# --------------- MZ expected correlations (Table 1)

# order of members: t1 s1 t2 s2

Rmz=matrix(0,4,4)

diag(Rmz) = 1

# MZ1 - MDZ2 (3,1)

rmz=TMZ=Rmz[1,3]=Rmz[3,1]=h2+c2+s2 #

# MZ1 - spouse1(2,1) MZ2 - spouse2 (4,3)

Rmz[1,2]=Rmz[2,1]=dp+s2*dy

Rmz[3,4]=Rmz[4,3]=dp+s2*dy

# cotwin spouse, MZ1 - spouse2, MZ2 - spouse 1 (4,1) (3,2)

Rmz[4,1] = Rmz[1,4] = Rmz[3,2] = Rmz[2,3] = TMZ*dp +s2*dy

# spouse 1 spouse 2 (4,2)

Rmz[4,2]=Rmz[2,4]= TMZ*dp2 + s2*dy2 + 2*s2*dy*dp

# --------------------------------------------------

# --------------- DZ expected correlations (Table 1)

# order of members: t1 s1 t2 s2

Rdz=matrix(0,4,4)

diag(Rdz) = 1

# DZ1 - DZ2 (3,1)

rdz=TDZ=Rdz[1,3]=Rdz[3,1]=.5*h2*(1+dp*h2) + c2 + s2

# DZ1 - spouse1(2,1) DZ2 - spouse2 (4,3)

Rdz[1,2]=Rdz[2,1]=dp+s2*dy

Rdz[3,4]=Rdz[4,3]=dp+s2*dy

# cotwin spouse, DZ1 - spouse2, DZ2 - spouse 1 (4,1) (3,2) #rCotwin-Spouse (DZs) = TDZ*dp + s2*dy

Rdz[4,1] = Rdz[1,4] = Rdz[3,2] = Rdz[2,3] = TDZ*dp + s2*dy

# spouse 1 spouse 2 (4,2)

Rdz[4,2]=Rdz[2,4]= TDZ*dp2 + s2*dy2 + 2*s2*dy*dp

# --------------------------------------------------

# from correlation matrices to covariance matrices

Smz=SD%*%Rmz%*%SD # covariance matrix

Mmz=rep(phmean,4) # means

Sdz=SD%*%Rdz%*%SD # covariance matrix

Mdz=rep(phmean,4) # means

# --------------------------- simulate data

cNmz=cNdz=1

# following only for exact data simulation

if (exact) {cNmz=(Nmz-1)/Nmz; cNdz=(Ndz-1)/Ndz}

datmz=mvrnorm(Nmz,mu=Mmz, Sigma=Smz/cNmz, emp=exact)

datdz=mvrnorm(Ndz,mu=Mdz, Sigma=Sdz/cNdz, emp=exact)

#

# write to external file?

if (wr) {

dat=matrix(2,Nmz+Ndz,5)

dat[1:Nmz,1]=1 # mz code 1

dat[1:Nmz,2:5]=datmz #

dat[(Nmz+1):(Nmz+Ndz),2:5]=datdz

write.table(dat,file="datj3",col.names=F, row.names=F)

}

#

# ---------------------------------------------- end simulation

colnames(datmz)=vnames=c('T1','S1','T2','S2')

colnames(datdz)=vnames=c('T1','S1','T2','S2')

datmz=as.data.frame(datmz) # mz dataframe

datdz=as.data.frame(datdz) # dz dataframe

# ------------------------------------------------ openmx spec

# modelDD = table 2 implementation

#

# NB: starting values for h,e,c,dm, ds (standardized matrices)

ModelDD = mxModel(model="DDM",

# parameters

mxMatrix(type="Full", nrow=1, ncol=1, free=TRUE, values=.5, label='path_h', name='h'), # add gen

mxMatrix(type="Full", nrow=1, ncol=1, free=TRUE, values=.5, label='path_e', name='e'), # env

mxMatrix(type="Full", nrow=1, ncol=1, free=TRUE, values=.1, label='s_am', name='dy'), # social am

mxMatrix(type="Full", nrow=1, ncol=1, free=TRUE, values=.3, label='path_c', name='c'), # shared c

mxMatrix(type="Full", nrow=1, ncol=1, free=TRUE, values=.1, label='path_s', name='s'), # Social

mxMatrix(type="Full", nrow=1, ncol=1, free=TRUE, values=.1, label='ph_am', name='dp'), # delta_mu

mxMatrix(type='Full', nrow=1, ncol=1, free=FALSE, values=1, label='o1', name='O1'),

#

# mxMatrix(type='Full',nrow=1, ncol=1, free=TRUE, label='vph', name='vPh'),

#

# derived

mxAlgebra(expression=e %*% e, name = 'e2'),

mxAlgebra(expression=h %*% h, name = 'h2'),

mxAlgebra(expression=c %*% c, name = 'c2'),

mxAlgebra(expression=s %*% s, name = 's2'),

mxAlgebra(expression=dy %*% dy, name = 'dy2'),

mxAlgebra(expression=dp %*% dp, name = 'dp2'),

#

#

# RBP 2006

#

#TMZ = rMZ = h2+s2+c2

#TDZ = rDZ = .5*h2*(1+dp*h2) + c2 + s2

#rSPOUSES = dp +s2*dy

#rCotwin-Spouse (MZs) = TMZ*dp + s2*dy

#rCotwin-Spouse (DZs) = TDZ*dp + s2*dy

#rSpouse1-Spouse2 (MZ) = TMZ*dp2 + s2*dy2 + 2*s2*dy*dp

#rSpouse1-Spouse2 (DZ) = TDZ*dp2 + s2*dy2 + 2*s2*dy*dp

#

mxAlgebra(expression=h2+s2+c2+e2,name='vph'),

mxConstraint(vph==1, name='c_stdvar'), # standardized

# correlation elements MZ - RBP 1996 table 1

mxAlgebra(expression=h2+c2+s2, name='rmzmz'), # # #TMZ = rMZ = h2+s2+c2

mxAlgebra(expression=dp+s2*dy, name='rmzsp'), ##rSPOUSES = dp +s2*dy

mxAlgebra(expression=rmzmz*dp+s2*dy, name='rmzctsp'), #rCotwin-Spouse (MZs)=TMZ*dp + s2*dy

mxAlgebra(expression=rmzmz*dp2 + s2*dy2 + 2*s2*dy*dp, name='rmzspsp'), # rSpouse1-Spouse2 (MZ) = TMZ*dp2 + s2*dy2 + 2*s2dy*dp

# correlation elements DZ - RBP 1996 table 1

mxAlgebra(expression=.5*h2*(1+dp*h2)+c2+s2, name='rdzdz'), ##TDZ = rDZ = .5*h2*(1+dp*h2) + c2

mxAlgebra(expression=dp+s2*dy, name='rdzsp'), # same as MZ #

mxAlgebra(expression=rdzdz*dp+s2*dy, name='rdzctsp'), #

mxAlgebra(expression=rdzdz*dp2 + s2*dy2 + 2*s2*dy*dp, name='rdzspsp') #

)

#

# starting values phm and phs (from cor to cov)

#

ModelDD_MZ = mxModel(model="DDMMZ",

mxData(observed=datmz, type='raw'),

mxMatrix(type="Full", nrow=1, ncol=4, free=TRUE, values=0, label=c('phm','phm','phm','phm'), name='mzphme'),

mxMatrix(type="Diag", nrow=4, ncol=4, free=TRUE, values=1, label=c('phs','phs','phs','phs'), name='mzphsd'),

mxAlgebra(expression=rbind(

cbind(1, DDM.rmzsp, DDM.rmzmz, DDM.rmzctsp),

cbind(DDM.rmzsp, 1, DDM.rmzctsp, DDM.rmzspsp),

cbind(DDM.rmzmz, DDM.rmzctsp, 1, DDM.rmzsp),

cbind(DDM.rmzctsp,DDM.rmzspsp, DDM.rmzsp, 1)),

name='mzcor'),

mxAlgebra(expression=mzphsd%*%mzcor%*%mzphsd, name='mzcov'),

# mxAlgebra(expression=DFM.vph%x%mzcor, name='mzcov'),

mxExpectationNormal(covariance='mzcov', means='mzphme', vnames),

mxFitFunctionML()

)

#

# starting values phm and phs should equal those in DFMDZ

#

ModelDD_DZ = mxModel(model="DDMDZ",

mxData(observed=datdz, type='raw'),

mxMatrix(type="Full", nrow=1, ncol=4, free=TRUE, values=0, label=c('phm','phm','phm','phm'), name='dzphme'),

mxMatrix(type="Diag", nrow=4, ncol=4, free=TRUE, values=1, label=c('phs','phs','phs','phs'), name='dzphsd'),

mxAlgebra(expression=rbind(

cbind(1, DDM.rdzsp, DDM.rdzdz, DDM.rdzctsp),

cbind(DDM.rdzsp, 1, DDM.rdzctsp, DDM.rdzspsp),

cbind(DDM.rdzdz, DDM.rdzctsp, 1, DDM.rdzsp),

cbind(DDM.rdzctsp,DDM.rdzspsp, DDM.rdzsp, 1)),

name='dzcor'),

mxAlgebra(expression=dzphsd%*%dzcor%*%dzphsd, name='dzcov'),

# mxAlgebra(expression=DFM.vph%x%dzcor, name='dzcov'),

mxExpectationNormal(covariance='dzcov', means='dzphme', vnames),

mxFitFunctionML()

)

#

cimx=mxCI(c('path_c','ph_am','path_s','s_am'))

# assemble

#

ModelDD_RBP06 = mxModel(model="DDM_RBP",ModelDD,ModelDD_MZ,ModelDD_DZ,cimx,

# old

# mxAlgebra(expression=DFMMZ.objective + DFMDZ.objective, name='DFfit'),

# mxFitFunctionAlgebra('DFfit')

# new way to do this

mxFitFunctionMultigroup( c("DDMMZ","DDMDZ") )

)

# run full 2006 model

#

# mxCheckIdentification(ModelDD_RBP06, details=FALSE)

#

r1DD = mxTryHard(ModelDD_RBP06, extraTries=50, interval=F)

#

#Ir1DD=round(cov2cor(vcov(r1DD)),5)

#

epar1=summary(r1DD)$parameters[1:6,5]

tpar1=c(h,e,dy,c,s,dp)

names(epar1)=names(tpar1)= c('h','e','dy','c','s','dp')

round(epar1,3)

round(tpar1,3)

#

# fit saturated model ................

r2Sat = mxRefModels(r1DD,run=TRUE)

mxCompare(r2Sat,r1DD) # if exact = T the likelihood ratio should be zero.

#

#

# test dm - phenotypic AM

#

ModelDD_RBP06_NOc=omxSetParameters(ModelDD_RBP06, labels=c('path_c'), values=c, free=FALSE)

r1DDNOc = mxRun(ModelDD_RBP06_NOc)

mxCompare(r1DD,r1DDNOc)

mxCompare(r2Sat,r1DDNOc)

epar2=summary(r1DDNOc)$parameters[1:5,5]

tpar2=c(h,e,dy,s,dp)

names(epar2)=names(tpar2)= c('h','e','dy','s','dp')

epar2

tpar2

#

ModelDD_RBP06_NOc=omxSetParameters(ModelDD_RBP06, labels=c('path_c'), values=0, free=FALSE)

r1DDNOc = mxRun(ModelDD_RBP06_NOc)

mxCompare(r1DD,r1DDNOc)

mxCompare(r2Sat,r1DDNOc)

epar3=summary(r1DDNOc)$parameters[1:5,5]

tpar3=c(h,e,dy,s,dp)

epar3

tpar3
